# MOrgAna: accessible quantitative analysis of organoids with machine learning

**DOI:** 10.1242/dev.199611

**Published:** 2021-09-07

**Authors:** Nicola Gritti, Jia Le Lim, Kerim Anlaş, Mallica Pandya, Germaine Aalderink, Guillermo Martínez-Ara, Vikas Trivedi

**Affiliations:** 1 European Molecular Biology Laboratory (EMBL), Barcelona 08003, Spain; 2 EMBL Heidelberg, Developmental Biology Unit, 69117 Heidelberg, Germany

**Keywords:** Fluorescence, Graphical user interface, Machine learning, Morphology, Organoids, Quantification

## Abstract

Recent years have seen a dramatic increase in the application of organoids to developmental biology, biomedical and translational studies. Organoids are large structures with high phenotypic complexity and are imaged on a wide range of platforms, from simple benchtop stereoscopes to high-content confocal-based imaging systems. The large volumes of images, resulting from hundreds of organoids cultured at once, are becoming increasingly difficult to inspect and interpret. Hence, there is a pressing demand for a coding-free, intuitive and scalable solution that analyses such image data in an automated yet rapid manner. Here, we present MOrgAna, a Python-based software that implements machine learning to segment images, quantify and visualize morphological and fluorescence information of organoids across hundreds of images, each with one object, within minutes. Although the MOrgAna interface is developed for users with little to no programming experience, its modular structure makes it a customizable package for advanced users. We showcase the versatility of MOrgAna on several *in vitro* systems, each imaged with a different microscope, thus demonstrating the wide applicability of the software to diverse organoid types and biomedical studies.

## Abstract (precis)

**Summary:** MOrgAna, an open access software with a user-friendly interface, implements deep learning networks to segment, quantify and visualize morphological and fluorescence information of hundreds of organoid images within minutes.

## INTRODUCTION

Organoids are cell aggregates capable of generating complex structures, thereby mimicking fully grown organs *in vitro* (Huch et al., 2017; Lancaster and Knoblich, 2014; Kretzschmar and Clevers, 2016). To develop such complex organization, organoids undergo highly dynamical processes, including growth, shape changes and emergence of gene expression patterns concomitant with cell fate commitments (Huch et al., 2013; van den Brink et al., 2014; Eiraku et al., 2011; Serra et al., 2019) These observables provide qualitative information on the patterning of organoids, and can be used to assess the wide variety of phenotypes displayed.

In recent years, due to novel engineering solutions and the need of buffering the large variability of organoid generation (Gritti et al., 2021), the number of experimental conditions have grown combinatorially and it is now possible to generate increasingly large datasets. There is therefore now a major bottleneck in the ability to inspect this huge number of images and quantify morphological and fluorescence parameters in space and time with high accuracy and in an unbiased manner. When quantitatively interpreted, such data have been crucial in dissecting the mechanisms responsible for organoid development (Phipson et al., 2019; Lukonin et al., 2020; Hof et al., 2021). Another hindrance is the limited access to an imaging system that can accommodate the variety of plates and devices used in organoid culture (Rossi et al., 2018). Generally, high-content screening (HCS) devices represent an ideal platform to image a large number of samples under a variety of conditions (Durens et al., 2020; Brandenberg et al., 2020; Vrij et al., 2016). However, there is a tradeoff between microscope availability, high-throughput capacity and image quality, often forcing researchers to use low-end stereoscopic microscopes that can suffice for qualitative assessment. Therefore, in order to extract quantitative information, much effort is required to adapt, if not rewrite, conventional algorithms to work around the signal to noise in images generated by the diverse devices used.

Nowadays, machine learning (ML)-based algorithms have become an essential tool across biomedical disciplines to perform a quantitative, unbiased analysis of microscopy images, and are either provided as part of proprietary software (e.g. MetaMorph, Imaris, Harmony and ZEN) or distributed open-source (e.g. FIJI, CellProfiler and ilastik); (Berg et al., 2019; Schindelin et al., 2012; Carpenter et al., 2006). In the context of HCS of 2D adherent cell culture, such software have been capable of, for example, identifying thousands of cells within wide fields of view and extracting biologically relevant features (McQuin et al., 2018). However, organoids display highly complex phenotypes, which are difficult to describe with traditional morphological features such as radius length, area, perimeter or average fluorescence intensity. Thus, standard high-throughput segmentation pipelines need to be adapted to detect individual large objects distributed among several images. Even though data-driven approaches to characterizing organoid phenotypes exist (Serra et al., 2019), so far little effort has been made to develop a user-friendly intuitive pipeline that can be used by a large community with limited programming experience.

**Fig. 1.**
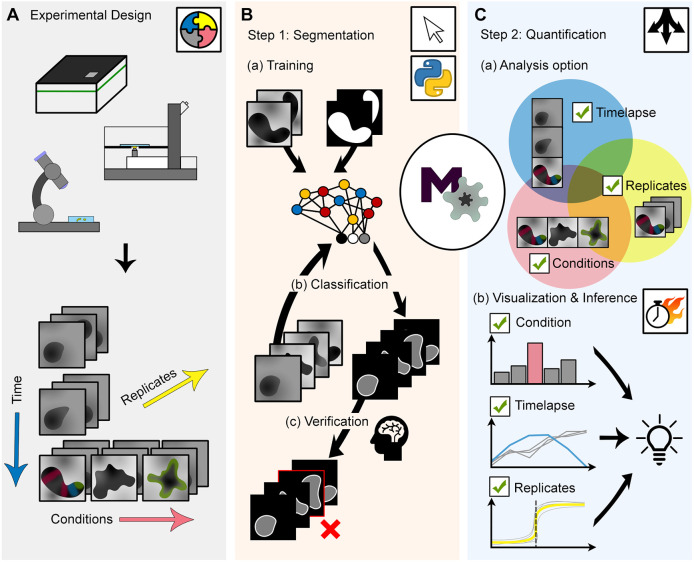
**MOrgAna workflow schematic.** (A) Versatility and flexibility of MOrgAna derives from its ability to accept, as input data, images acquired by diverse devices such as high-content screening devices, confocal microscopes and simple benchtop stereo-microscopes, and under diverse experimental scenarios such as those acquired over the course of several days, across experimental replicates or under different perturbation conditions. (B) Schematic of the segmentation workflow of the Python-based MOrgAna. Users first create binary masks of a few representative images for training of an image segmentation model. With the trained network, additional masks of unseen images can be easily generated and subsequently checked and modified manually, all of which can be accomplished with a few mouse clicks through its graphical user interface. (C) With binary masks and input images, users can then cluster their images into different groups and choose the method of image analysis based on their data type. The modular nature of MOrgAna allows the user to build an analysis pipeline by combining options for timelapse, replicates or conditions depending on the experimental scenario in A. MOrgAna quickly produces quantitative plots based on morphological and fluorescence parameters, thereby visually answering the users’ research questions, leading to biological discoveries.

In this work, we present MOrgAna, a Machine-learning-based Organoid Analysis software. MOrgAna is a Python package that implements an easy-to-use ML pipeline to segment hundreds of organoids, each fully contained in a single 2D image, within minutes (Fig. 1A,B; Fig. S1). MOrgAna provides a simple, yet powerful, visualization and quantification toolbox for morphological as well as fluorescence information (Fig. 1C; Fig. S2). We proved the flexibility of MOrgAna by applying its pipeline to characterize morphological and fluorescence parameters for several sample types, including human brain organoids (Lancaster et al., 2017), zebrafish explants (pescoids) (Fulton et al., 2020), mouse embryonic organoids (gastruloids) (van den Brink et al., 2014) and intestinal organoids (Serra et al., 2019). The images were acquired with different microscopy devices, magnifications and fields of view, thus demonstrating the wide applicability of MOrgAna (Table S1). For users without coding experience, MOrgAna can run through a graphical user interface (GUI) to quickly provide visual quantitative graphs (Fig. S2). For more experienced users, we additionally provide ready-to-use code in the form of Jupyter notebooks (Materials and Methods) that can be customized at will and integrated into advanced image analysis pipelines.

## RESULTS AND DISCUSSION

To provide a widely applicable tool, we developed a pipeline that is divided into two separate and independent parts: the first segmentation step is based on the bright field image of the organoid (Fig. 1B), followed by a quantification step that takes into account all bright field and fluorescence channels available (Fig. 1C). The rationale behind this design is that bright field images are usually readily available when performing any imaging experiment, whereas fluorescence signals depend on labeled structures within organoids, and are thus more difficult to use for segmentation purposes. Moreover, especially in biomedical applications, patient-derived organoids are typically monitored in bright-field mode and fluorescently labeled at the end of the experiment.

To define the training set, features of pixels in provided images are computed with morphological parameters, such as the widths of Gaussian and Laplacian filters, which are initialized to default values and can optionally be modified by the user (Materials and Methods). In most applications, we found that our default parameters, generating 18 features per pixel, provide sufficient information for successful object segmentation. To improve flexibility and applicability, as an alternative to training image segmentation networks based on Logistic Regression (classical ML), MOrgAna can also use Multi Layer Perceptron with two hidden layers (deep learning algorithm) to recognize organoids. As development of organoids generally happens over several days and requires media exchange, aggregates can be surrounded by delaminating cells and debris, making it complicated to accurately detect their boundaries. Therefore, the networks in MOrgAna learn to classify pixels into three classes: background, organoid and organoid edge. The resulting trained network can then be applied to previously unseen images. Although the standard classifier network performs well in most cases, MOrgAna additionally and automatically predicts masks using the watershed algorithm, thus providing the user with an alternative segmentation option to choose from. Importantly, MOrgAna presents a manual curation step, whereby the user is prompted to inspect, detect and correct segmentation errors or to ignore corrupted images. Therein, a final segmentation mask of the organoid within the image is computed and stored (Fig. 1B). When multiple organoids per image exist and a segmentation can be generated by simple thresholding or using third party software, users can import the image and masks. MOrgAna will then save individual objects into separate files together with their masks. These can be used to perform a finer segmentation of the organoids or to proceed with the quantification pipeline using the previously provided masks (Fig. S3).

Upon completion of the segmentation task, MOrgAna is then equipped to compute and visualize several morphological and fluorescence features characteristic of organoid development (Fig. 1C). For this aim, we created a simple, yet powerful, pipeline that performs the computations and retrieves the results for future visualizations in a format that is modular and can be adapted to the experiment type. For example, data from different experimental replicates and conditions can be clustered into separate groups, while parameters among different conditions can be compared. On the other hand, data obtained from time lapse experiments are analyzed with an additional time dimension. Time lapse trajectories from different replicates and conditions can also be grouped and shown together in one plot. The range of possibilities available, as well as a detailed description of the interface, are thoroughly described at the online MOrgAna repository (see Materials and Methods).

To first test the performance of the MOrgAna segmentation module and benchmark it with existing pipelines, we used a dataset consisting of 91 organoid images (Fig. S4A). The images were manually annotated to provide a ground truth reference with which to compare the segmentation results. We chose to use CellProfiler (Carpenter et al., 2006) and OrganoSeg (Borten et al., 2018) for comparisons as they are the two popular tools used for automated image analysis, with the second being organoid-specific and using local adaptive thresholding for segmentation (Fig. S4B-D). We compared the segmentation results from all three software packages against the ground truth images based on standard metrics such as Jaccard distance, precision and accuracy, and found that MOrgAna performed better than both CellProfiler and OrganoSeg (Fig. S4E). We would like to emphasize that, although the chosen existing pipelines use standard morphological operations to segment the images, MOrgAna uses ML algorithms, which are expected to perform better as they directly learn a non-linear relationship between input images and the provided ground truth. Moreover, thanks to the fact that MOrgAna classifies pixels into three classes, we observed a higher accuracy in segmenting complex organoid boundaries. With respect to the run time, we observed that, although OrganoSeg is comparable with MOrgAna, CellProfiler processing time was more than twice as long (Fig. S4F).

**Fig. 2.**
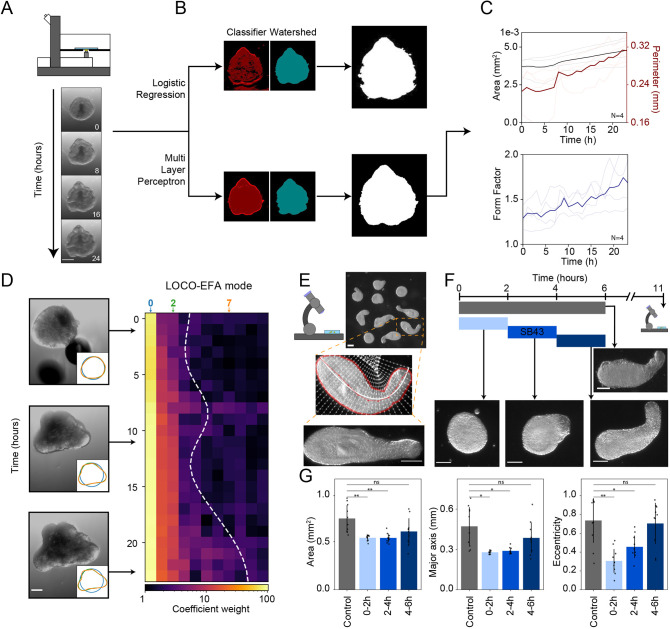
**Morphology quantification of organoids over time and across replicates with MOrgAna.** (A) Representative time-lapse images of a 7 day human induced pluripotent stem cell-derived brain organoid acquired on a confocal microscope depicted in the schematic on top. (B) Segmentation output of a single brain organoid with Logistic Regression (top) and Multi Layer Perceptron (bottom) models. Left masks are obtained with the classifier (red) and the watershed algorithm (blue). Binary images represent the final masks after postprocessing. (C) Temporal dynamics of the area, perimeter and form factor for brain organoids (*n*=4) over the course of 24 h of development. (D) LOCO-EFA quantification for a single brain organoid. Insets represent the shape reconstructed using 0 (blue), 2 (green) and 7 (orange) ellipse modes. Kymograph represents the weight of every ellipse mode (log scale). Dashed white line labels the highest mode necessary to describe 95% of the shape. (E) Representative image of pescoids as obtained from a dissection microscope. Enlarged is a single pescoid: red line represents the edge of the segmented mask, white line the computational midline and white dots a subsample of the meshgrid used to computationally straighten the organoid (bottom image). (F) Schematic of the inhibition experiment with representative images of control and inhibited pescoids at 11 hours post fertilization. (G) Morphological quantification and comparison between control (gray) and pescoids inhibited at the time window highlighted in F. Data are mean±s.d., *n*=10 for all conditions shown. *P<0.05, **P<0.01 (using a Welch two sample *t*-test). ns, not significant. Scale bars: 200 µm (A,D); 100 µm (E,F).

Next, we tested the ability of MOrgAna to detect global morphological changes in time lapse images. We chose to analyze confocal microscopy images of human brain organoids (Lancaster et al., 2017), cell aggregates that display neuroectoderm formation associated with complex morphological changes. Brain organoids start as spherical aggregates and grow rapidly in size to form irregular structures displaying several lobes (Fig. 2A). We observed that MOrgAna is capable of segmenting and quantifying subtle changes in their morphological shape, including area, perimeter and form factor (Andriankaja et al., 2012) (Fig. 2B,C). To better characterize the phenotypic complexity observed, we additionally implemented lobe contribution elliptical Fourier analysis (LOCO-EFA), a descriptor typically used to quantify the shape of tissues and cells (Andriankaja et al., 2012; Samuel et al., 2018). LOCO-EFA represents a powerful algorithm that decomposes the object outline into a series of coefficients related to the elliptical fundamental modes needed to accurately describe the lobes (Sánchez-Corrales et al., 2018). This is particularly useful in cases where conventional parameters based on fit to an ellipse such as eccentricity, major and minor axes, cannot capture the complexity of the organoid shape. We applied LOCO-EFA to time lapse images of the human brain and intestinal organoids (Fig. 2D; Fig. S5A,A′), and observed that the coefficients accurately capture the appearance of lobes, and that contributions from higher modes are necessary to describe the increasingly complex shapes developed by the organoids (Fig. S5B,B′). In addition, we observed that the highest non-negligible mode roughly corresponds to the number of lobes present in the organoids, as previously described (Sánchez-Corrales et al., 2018).

LOCO-EFA is a powerful tool in describing shapes of amorphous aggregates; however, it is not ideal when morphological changes result from the curving of the body axis. In such cases, higher elliptical modes could be biased by the overall curved organoid shape, resulting in bent organoids being incorrectly assigned higher coefficient values than straight ones. This happens, for example, in zebrafish explants known as pescoids, which break their original spherical symmetry and form elongated and curved structures with an anteroposterior (AP) pattern over the course of 11 h (Fig. 2E) (Fulton et al., 2020; Schauer et al., 2020). In such cases, MOrgAna is equipped with an algorithm to extract a morphological midline of the aggregates purely based on the binarized mask. This midline is then used to computationally straighten the binarized images (Fig. 2E; see also Materials and Methods) and re-compute all morphological parameters, which now also include eccentricity and the length of the major and minor axes. Using this straightening approach, MOrgAna is capable of detecting subtle morphological changes induced by experimental perturbations. When imaged at the time of maximum elongation, we found that pescoid development was affected by different durations of exposure to a small molecule inhibitor of Nodal signaling (Fig. 2F,G).

**Fig. 3.**
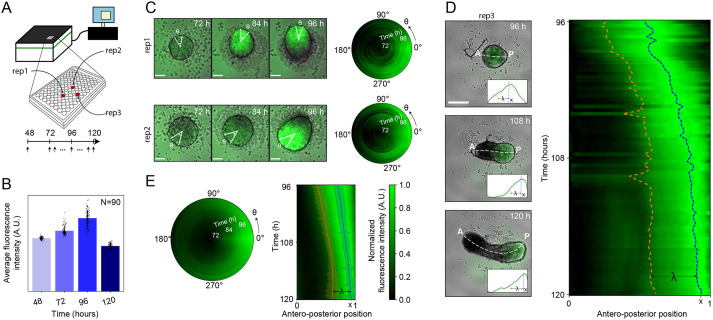
**Fluorescence quantification of high-throughput image datasets with MOrgAna.** (A) Schematic of a typical high-throughput imaging experiment, exemplified here using gastruloids cultured in a 96-well plate and imaged on a high-content screening device at 48 h and then continuously for 2 days between 72 and 120 h post aggregation. (B) Average fluorescence intensity for gastruloids (*n*=90) at 24 h intervals. (C) Representative images of two gastruloids between 72 and 96 h post aggregation, with the angular profile shown as an angular heatmap. The 0 angle is defined as the direction of maximal radial fluorescence intensity. (D) Example images of a gastruloid at 96, 108 and 120 h post aggregation. Dashed white line shows the computational midline. A, anterior; P, posterior. Insets show anteroposterior (AP) profile and exponential fit for the example images. Kymograph represents the AP profile at all imaged timepoints for a single gastruloid. Dashed blue and orange lines represent the position of the highest brachyury expression and the position corresponding to its decay length, respectively. (E) Average angular and AP profiles for *n*=39 and *n*=90 gastruloids, respectively. Blue and orange bands represent the standard deviation of the peak position and decay length, respectively. Scale bars: 100 μm.

Besides morphological features, fluorescently labeled organoids provide valuable information on the underlying patterns of differentiation and lineage commitments. Such informative fluorescence intensities can be quantified using MOrgAna. Besides analysis of standard average and background intensities, MOrgAna can also incorporate the spatial context through the computationally straightened mask to determine the fluorescence intensity profiles along different axes depending on the shape and the biological identity of different regions within the organoids such as the longitudinal (AP), orthogonal (mediolateral), angular and radial directions. To test the ability of MOrgAna to quantify and visualize these profiles, we used images of gastruloids, aggregates of mouse embryonic stem cells (mESCs) that have been shown to mimic the early stages of mammalian embryonic development and patterning (van den Brink, 2014). Gastruloids were generated in 96-well plates and imaged on an HCS device (Fig. 3A). Gastruloids start as spherical aggregates that, over the course of 5 days, develop into elongated structures with the establishment of the three main body axes: AP, mediolateral and dorsoventral (DV) (Beccari et al., 2018). These events are accompanied by cellular transcriptional changes that establish a characteristic posterior domain expressing the mesodermal marker brachyury (Bra; Turner et al., 2017), which was fluorescently labeled in the cell line used (Fig. 3B,C). Quantification of average fluorescence intensities in the gastruloids with MOrgAna (Fig. 3B) revealed a gradual increase in brachyury expression during the initial growth phase and a subsequent signal decrease during the elongation phase (Fig. 3B). Between 72 and 96 h after aggregation, brachyury starts being expressed homogeneously throughout the gastruloids and eventually becomes restricted to one side of the spherical aggregate, a polarization event that is thought to drive gastruloid patterning. This development is well captured with the MOrgAna angular profile quantification (Fig. 3C). On the following day, gastruloids elongate dramatically, with the brachyury domain being confined to the posterior-most side of the gastruloids, a patterning dynamics that is well described by the AP profile quantification of MOrgAna (Fig. 3D).

To test the quantification along the radial direction, we analyzed previously published time lapse images of intestinal organoids (Serra et al., 2019), which undergo a spherically symmetrical growth with occasional crypts rather than a stereotypical AP elongation (Fig. S5C,C′). Finally, we performed the full segmentation and fluorescence quantification analysis pipeline for a time-lapse dataset with over 90 gastruloids and 144 timepoints, thus testing the scalability of the MOrgAna pipeline and displaying its applicability to large datasets (∼0.5 TB, Fig. 3E). In particular, we quantified the average angular and AP profiles of brachyury expression and computed the relative position of the expression peak and the normalized length of the AP profile. In total, using only ∼1% of the total number of images to train a ML network, the ∼0.5 TB of data that formed the gastruloid time-lapse dataset was analyzed within a few hours and the output graphs were generated on the same day as processing.

In conclusion, we developed MOrgAna, a user-friendly and modular segmentation and quantification pipeline, proving its applicability to the analysis of several organoid types and imaging devices. This was inspired by previous works that quantified embryonic organoid images with custom scripts (Turner et al., 2017; Moris et al., 2020) but that were crucially limited to considerable manual annotation and one organoid type. The MOrgAna pipeline is capable of processing hundreds of 2D images within minutes, reliably extracting morphological and fluorescence information and providing visual quantitative graphs for the user to interpret in the context of the biological question of interest. In the case of tiled acquisition of large organoids, postprocessing of the images is necessary before it can be used as input of the MOrgAna pipeline. Such postprocessing includes, but is not limited to: stitching, correction for inhomogeneous illumination of the sample, denoising and deconvolution. Many excellent solutions for these tasks already exist, such as the FIJI plugin BigStitcher (stitching), Noise2Void (denoising) and DeconvolutionLab2 (deconvolution) (Hörl et al., 2019; Krull et al., 2019 preprint; Sage et al., 2017) The quantitative results are saved as JavaScript Object Notation (json) files, which can be accessed with common text editors. In addition, the data for every graph can be saved in csv or xls files and therefore can serve as input for other visualization programs. Owing to its user friendly GUI and Python implementation (Figs S1 and S2), MOrgAna can be used by users with little to no programming experience as well as by more experienced users as part of more advanced image analysis pipelines (Oriola et al., 2020 preprint). In particular, our Jupyter Notebooks provide a ready-to-use platform to, for example, compute and visualize curve-fitting parameters of the fluorescence intensity profiles (Fig. 3D,E). Together, these features make MOrgAna a useful tool for researchers in a wide variety of organoid-based imaging experiments and we expect it to become widely used to perform image quantification in an automated, quick and standardized manner.

The field of image and shape analysis is continuously growing; therefore, we intend to maintain and expand the current version of the software to integrate more complex shape descriptors to further characterize the phenotypic complexity of organoids. Importantly, the modular nature of the MOrgAna code can facilitate its integration with existing frameworks, such as CellProfiler, in future. Such efforts will allow the establishment of flexible and accessible image analysis pipelines for users. Similarly, as the characterization of organoid phenotypes is being driven by the study of their constituent single cells, it can be envisioned that modules to segment and quantify secondary objects within organoid images will be useful in future. Upon acquisition of diverse datasets taken by different imaging devices in the field, MOrgAna will subsequently become equipped with pre-trained, more complex and fully convolutional deep learning networks that can be directly applied to previously unseen images, thus further reducing the gap between microscopy images and biological discoveries.

## MATERIALS AND METHODS

### Sample preparation and image acquisition

As previously described (Anlas et al., 2021), we used a Bra:GFP transcriptional reporter cell line (Gadue et al., 2006; Fehling et al., 2003) which was shown to faithfully reproduce the expression of brachyury in a variety of differentiation conditions (Turner et al., 2014). Cells were maintained in ES-Lif (ESL), consisting of KnockOut D-MEM supplemented with 10% fetal bovine serum (FBS), 1× non-essential amino acids (NEEA), 50 U/ml Pen/Strep, 1× GlutaMax, 1× sodium pyruvate, 50 μM 2-mercaptoethanol and leukemia inhibitory factor (LIF, homemade) in 0.1% gelatin-coated (Millipore, ES-006-B) tissue culture 25 cm^2^ flasks (T25 flasks, Corning 353108) at 37°C and 5% CO_2_. ESL was prepared and provided by the Tissue Engineering Unit of the Centre for Genomic Regulation (CRG; Barcelona, Spain). Briefly, cells were grown up to 50-70% confluency before being trypsinized and centrifuged at 180 ***g***. We then added 40 μl of the resulting cell suspension in differentiation medium N2B27 (Ndiff 227, Takara, Y40002) to each well in a U-bottom, low adhesion 96-well plate (96WP, Greiner, 650970), corresponding to ∼300 cells per well. An additional 150 μl of N2B27 with 3 μM CHIR99 (Sigma-Aldrich, SML1046) was added into each well after 48 h, with daily N2B27 medium exchange after 72 h. Gastruloids were imaged every 24 h with the Opera Phenix High Content Screening System (PerkinElmer) in wide-field mode with 10× magnification (NA 0.3, air objective). It is worth noting that a few gastruloids are usually lost during the daily media exchange or discarded because of insufficient elongation caused by media evaporation, particularly in the wells located along the edges of the plate. For this reason, the typical sample size is ∼90 gastruloids per multi-well plate. For the time-lapse dataset, images of gastruloids generated in five of the eight columns were acquired every 20 min, with gastruloids maintained at 37°C, 5% CO_2_.

Pescoids were generated as detailed in Fulton et al. (2020). Zebrafish embryos were grown at 28.5°C until the 256 cell stage (∼2.5 h after fertilization). Next, embryonic cells were severed from the embryo using an eyelash tool. The explants were allowed to compact for ∼1 min before being transferred to a 60 mm×20 mm Petri dish (Sigma-Aldrich) containing 8 ml of Leibovitz's L15 Medium (Thermo Fisher Scientific, 11415049) with 3% FBS and 1% Pen/Strep. Culture medium was changed after ∼6 h. Bright-field images were taken on a benchtop Leica stereomicroscope S9i ∼11 h after pescoid generation. For perturbation experiments, pescoids were exposed to the small molecule SB43 (Sigma-Aldrich, #616461), a potent TGF-β inhibitor, for different 2 h time windows.

Brain organoids were prepared from human induced pluripotent stem cell line iPS(IMR90)-4 cultured in STEMFlex medium (Thermo Fisher Scientific), and generated using STEMdiff™ Cerebral Organoid Kit (Stemcell Technologies, 08570), including modifications from Lancaster et al. (2017). Brain organoids were imaged between 7 and 8 days after preparation on an Olympus FV3000 confocal microscope, using a 10× objective (NA 0.4) with brightfield. As brain organoids are large structures which spread over several focal planes, 3D image stacks were acquired and the average stack projections were subsequently used for image analysis.

### Benchmarking with established segmentation algorithms

To benchmark the performance of the MOrgAna segmentation module, we used two popular automated image segmentation pipelines: CellProfiler and OrganoSeg (Carpenter et al., 2006; Borten et al., 2018) and a dataset composed of 91 organoid images (Fig. S4A). For MOrgAna, we used three images (∼3% of the available images) to train the ML network and directly applied it to the remaining images in the dataset using default settings. For CellProfiler segmentation, we used the following pipeline. First, images were smoothed using morphological Opening and Closing with a structuring element of 25 pixel diameter. Image intensities were inverted with the ImageMath module and Primary objects were identified using Global Otsu with two classes as a thresholding method. Finally, to filter out debris, only the largest identified object was considered (MeasureObjectSizeShape followed by FilterObjects). For OrganoSeg, we used the default pipeline with Intensity Threshold=0.5, Window Size=500 and Size Threshold=5000. Similar to CellProfiler, we removed debris by filtering objects smaller than the largest identified object in the image. These analysis-generated masks for all the 91 organoids were then analyzed (Fig. S4A-D).

To perform a quantitative analysis, we used a custom Python script to linearize the mask into a 1D array and compute the Jaccard distance, defined as:
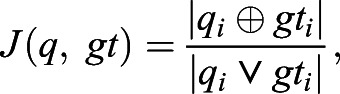
where 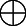 and ∨ indicate the logical XOR and AND operators. In addition we computed the precision (*P*) and accuracy (*A*), following the definitions:
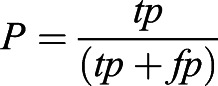

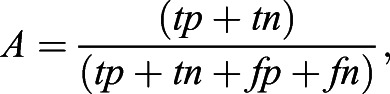
where *tp*, *tn*, *fp* and *fn* represent true positive, true negative, false positive and false negative pixel classification, respectively (Fig. S4E). Finally, we computed the runtime by manually monitoring the starting and finishing time of processing for every pipeline (Fig. S4F).

All processing pipelines used (including the CellProfiler project, the custom script used to filter the OrganoSeg masks and the Python code used to generate the quantitative measures) are available at the GitHub repository (Benchmarking, https://github.com/LabTrivedi/MOrgAna.git).

### Statistical analysis

Statistical analysis for both pescoid inhibition and benchmarking of MOrgAna was conducted with custom code written in RStudio Version 3.6.1. In the case of the pescoid data, the differences between conditions were evaluated using a *t*-test. Normality and homogeneity of variance were tested using Shapiro and Bartlett tests, respectively. As homogeneity of variance was discarded, we used the var=FALSE option in the *t*-test function, thus employing the Welch Two Sample *t*-test. As multiple statistical tests were performed, we applied the Bonferroni correction. In the case of benchmarking with CellProfiler and OrganoSeg, thanks to the large number of samples (*n*=91), we tested the differences between the pipelines using the single-step multi-comparison statistical Tukey test.

### Data handling and segmentation

Input images for the MOrgAna pipeline are multi-channel TIF files, with the first channel being used for the segmentation (typically the bright-field image). As common high-content screening systems output one file per channel, we provide a FIJI macro (found in the MOrgAna GitHub repository), to compile the individual files into the image format required for MOrgAna.

To train an image segmentation model, a few representative images are copied into a ‘training set’ subfolder. During the training of the model, images can optionally be resized to reduce computation time. Next, users can choose to use one of two feature extraction modes: ‘Ilastik’ or ‘Daisy’. The first includes Gaussian, Laplacian, gradient magnitude filters and difference of Gaussian computed for different filter widths (σ=1, 2, 5, 15 pixels), similar to previously established segmentation pipelines (Berg et al., 2019). Choosing the ‘Daisy’ option, a large number of texture features are added to the computation (Tola et al., 2010), which are extracted using the implementation from the scikit-image Python package (van der Walt, 2014).

Previously generated binary masks of the ‘training set’ images are subsequently dilated to include boundary regions of organoids in the final ground truth images. The pixels in training images are therefore identified as either background, organoid proper or organoid edge. A fraction of pixels (set by default to 50%) within the input images are extracted for training. Furthermore, there exists an option to define an extraction probability bias, which is useful when organoids occupy a small region of the full input image, and a random distribution of pixels would result in a training set heavily biased towards background pixels. For training, we employed either Logistic Regression or Multi Layer Perceptron models from the Python package scikit-learn (Pedregosa et al., 2011) with higher weights assigned to organoid edge pixels. Once trained, the model and the parameters used for feature extraction and training are stored in csv files in the model folder.

Next, trained models can be used to predict segmentations of previously unseen images. For this purpose, multi-channel TIF files in the same folder can be loaded and, with the ‘Generate Masks’ button, extraction and prediction with the same parameters used for the model training are conducted for these additional images. Then, pixels within input images are assigned probabilities of belonging to the three classes: background, organoid proper and organoid edge. A classifier mask is generated by assigning each pixel the class with the highest probability. Concurrently, a watershed algorithm is used to perform a second segmentation. In this algorithm, the edge probability map is used as input and the center of mass of the organoid proper map as seed. Both classifier- and watershed-based masks generated are saved.

Manual inspection is then required to generate the final mask. By selecting ‘Inspect Images’, users can quickly inspect and select the classifier- or watershed-based mask or choose to make another manually. With the mask selected, final binary images are generated by standard morphological operations such as filling holes, removing small objects and dilations. In particular, objects smaller than the largest detected object and any additional object touching the edge of the image are discarded. This efficiently allows debris and organoids that partially appear in the same field of view to be discarded. Parameters of these operations can be manually modified to the users’ satisfaction in the same inspection window.

After generation of the final masks, morphological and fluorescence intensity parameters are computed when the corresponding graphs are called by the user. In the quantification tab, morphological information can be obtained for both the ‘unprocessed’ and ‘straightened’ binary image. The latter is computed based on a distance transform map and skeletonization of the mask. Distance transform typically works well at the center of the object but generates multiple branches at the poles of the binarized mask; therefore, only the skeleton between the two closest connected points is kept and extended along the tangential direction to the edge of the organoid. We thus obtained the anchor points along which the full midline and meshgrid are determined and used for computational straightening.

All morphological and fluorescence intensity information are saved as json files. In addition, after generating a graph, the user can adjust pixel size to obtain absolute values of dimensionality measures such as perimeter and area, perform statistical analysis between conditions and save the data into csv or xls files, thus they can be retrieved for future inspection and as input in other processing pipelines.

### Decay length estimation

To estimate the decay length of T/Bra expression along the AP direction, we assumed a 1D model in which a point source is confined at a location x_0_ within the AP domain. We further assumed impermeable boundary conditions (zero flux at either ends x=0 and x=L, where L is the length of the gastruloid). The solution for this simple 1D model is
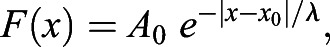
where A_0_ represents the production at the source and λ is the decay length of the fluorescence gradient profile relative to its source position x_0_. Individual timepoints from every gastruloid dataset were analyzed separately. Next, average and standard deviation of the decay length at every time point were computed.

### Software availability

MOrgAna is freely available as a GitHub repository at https://github.com/LabTrivedi/MOrgAna.git, and as a Python package that can be installed with the widely used PyPi library.

## Supplementary Material

Supplementary information
