# Supplementary material for: MOrgAna: accessible quantitative analysis of organoids with machine learning
Source: Development. 2021 Sep 7;148(18):dev199611. doi: 10.1242/dev.199611 (PMC8451065; doi:10.1242/dev.199611)
Supplement: Supplementary information [file develop-148-199611-s1.pdf]

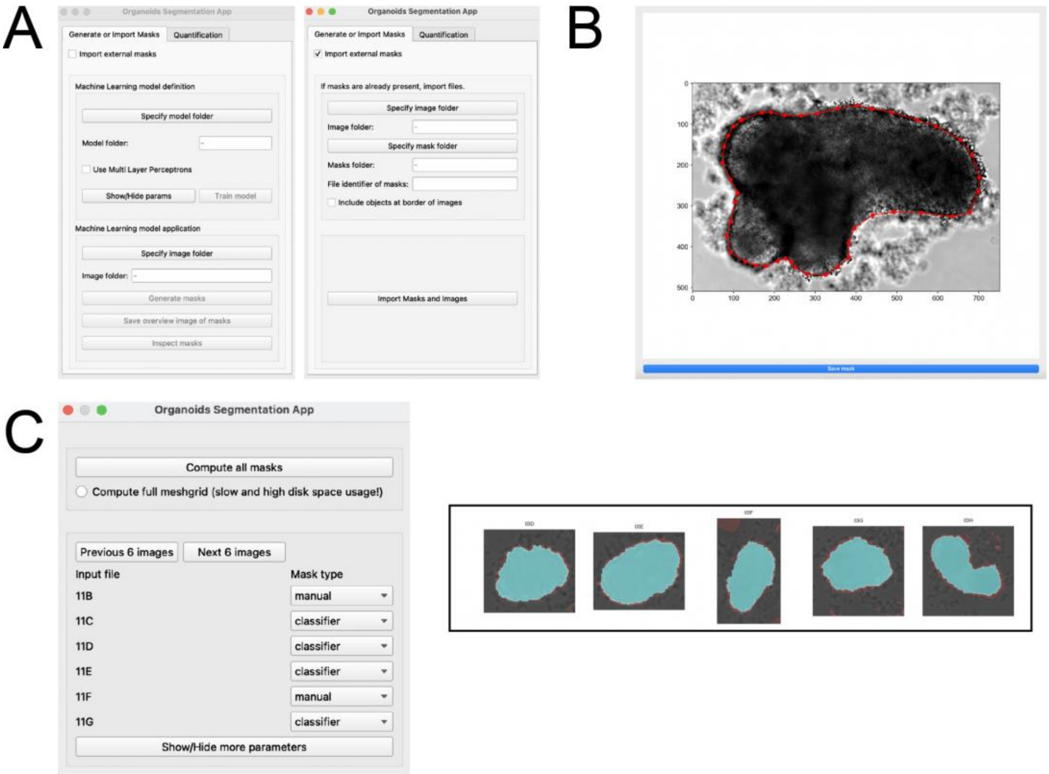

Figure S1. Graphical user interface of *MOrgAna*: input images and segmentation. A) Model definition and segmentation section (left) and module to import previously segmented datasets (right). B) Example of a bright field image of an organoid which is presented to the user for manual annotation of the ground truth binary mask (red line). C) Prediction panel (left) and manual inspection (right).

Development • Supplementary information

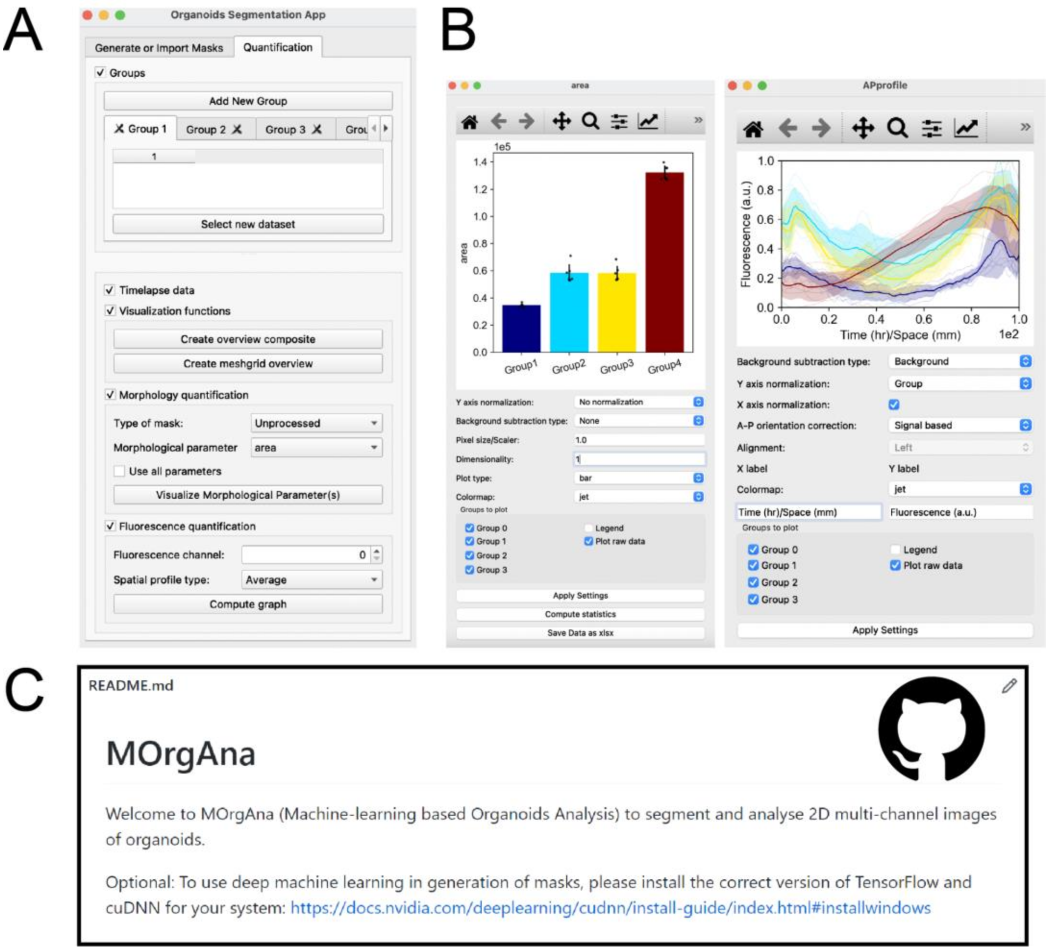

Figure S2. Graphical user interface of *MOrgAna* and GitHub page: quantification and output. A) Panel to cluster different datasets into separate “groups” for future visualization and comparison. Datasets can be treated as time lapse (tick box). Overview images can be generated with the “Visualization functions” buttons. B) Morphology (left) and Fluorescence intensity (right) visualization graphs can be generated and datasets from different groups easily compared. C) The GitHub repository at which all open-source MOrgAna code is found. In the same repository, advanced users can find ready-to-use Jupyter Notebooks.

Development • Supplementary information

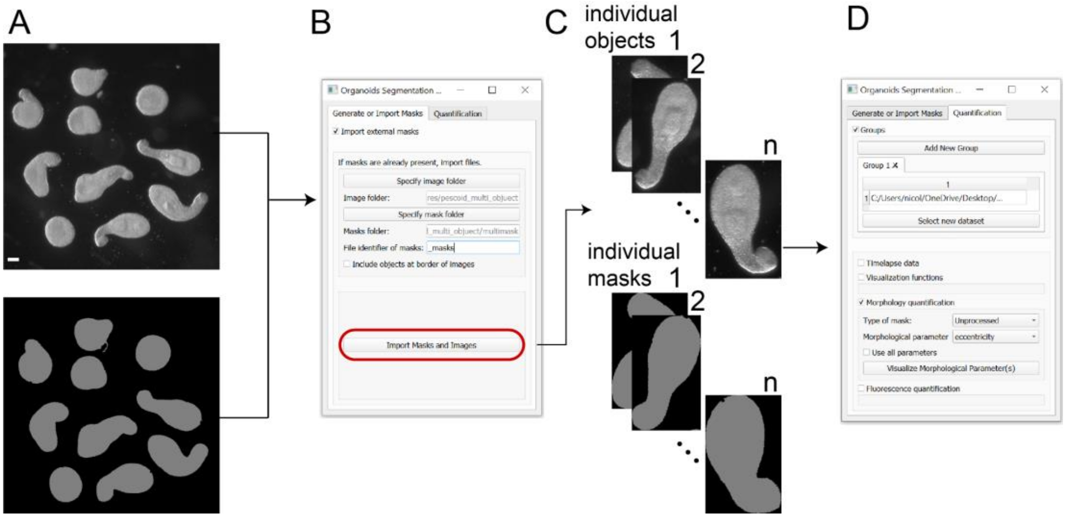

Figure S3. MOrgAna pipeline for pre-segmented object parsing. A) A single image containing multiple objects and previously segmented with OrganoSeg. Scale bar: 100 µm. B) The raw image and the masks can be imported in MOrgAna using the parsing functionality. C) Single objects are automatically stored in individual files and their masks are parsed accordingly. D) The resulting data structure can be directly used for the subsequent quantification pipeline of MOrgAna.

Development • Supplementary information

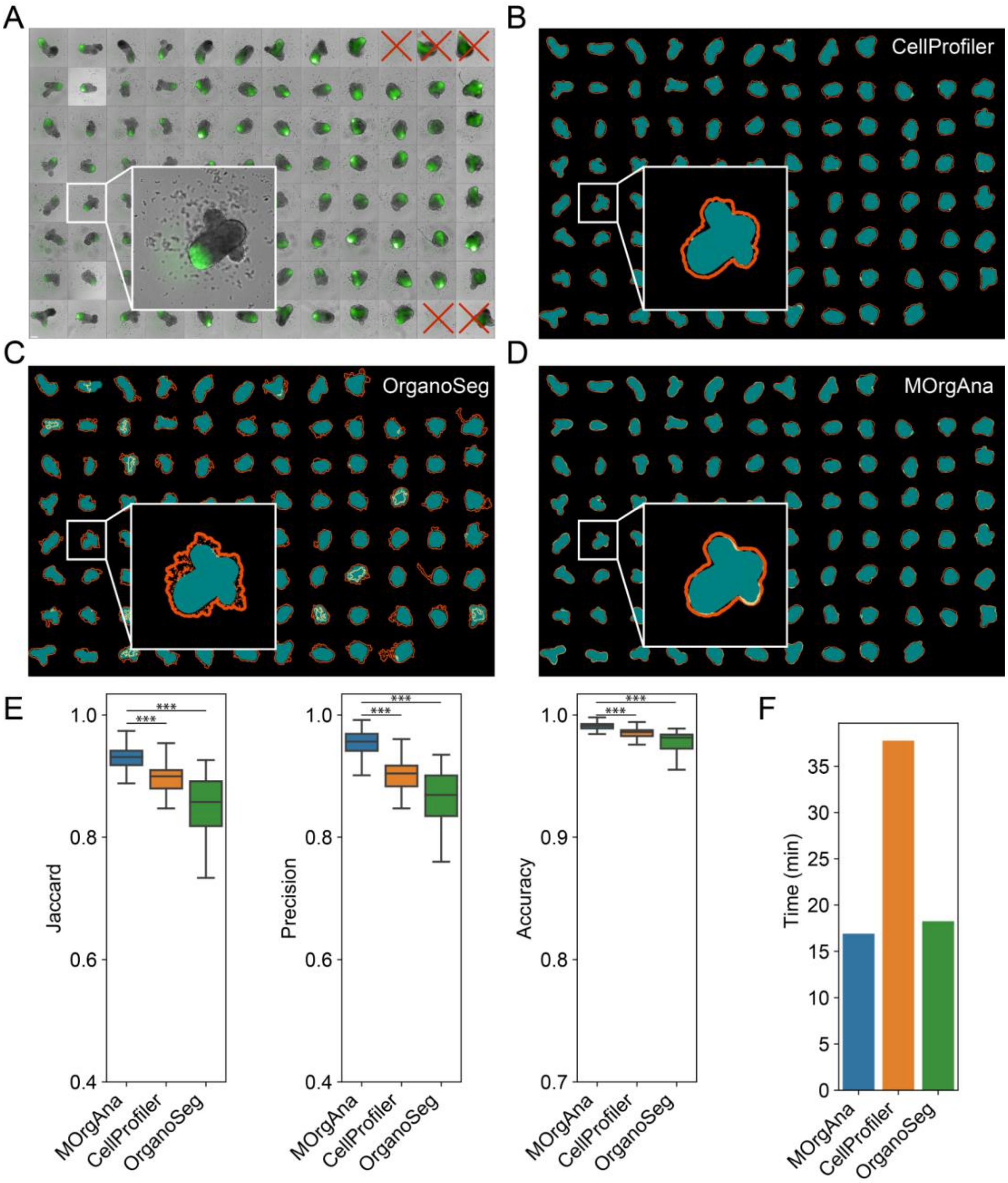

Figure S4. Benchmarking of MOrgAna with existing image analysis pipelines. A) Dataset used to benchmark MOrgAna with CellProfiler and OrganoSeg consists of gastruloids generated in a 96 wells plate and imaged on a high content screening device. Five of the 96 wells were discarded because no organoid was present or because it was not fully contained in the field of view (indicated by red crosses), thus resulting in a dataset of 91 gastruloid images. Scale bar: 100 um. B-D) Overlay of the manual annotation used as ground truth (cyan) and the edges for the segmentations generated with CellProfiler (B), OrganoSeg (C) and MOrgAna (D) (dark orange). Yellow lines represent boundaries within the ground truth mask, that is, cases in which the mask contains holes. In (A)-(D), inset highlights the raw image and the segmentation results. E) Quantitative measures used to compare the three segmentation methods include Jaccard distance, Precision and Accuracy. (*** p-value<0.001). F) Comparison of the run time for the three pipelines, in minutes.

Development • Supplementary information

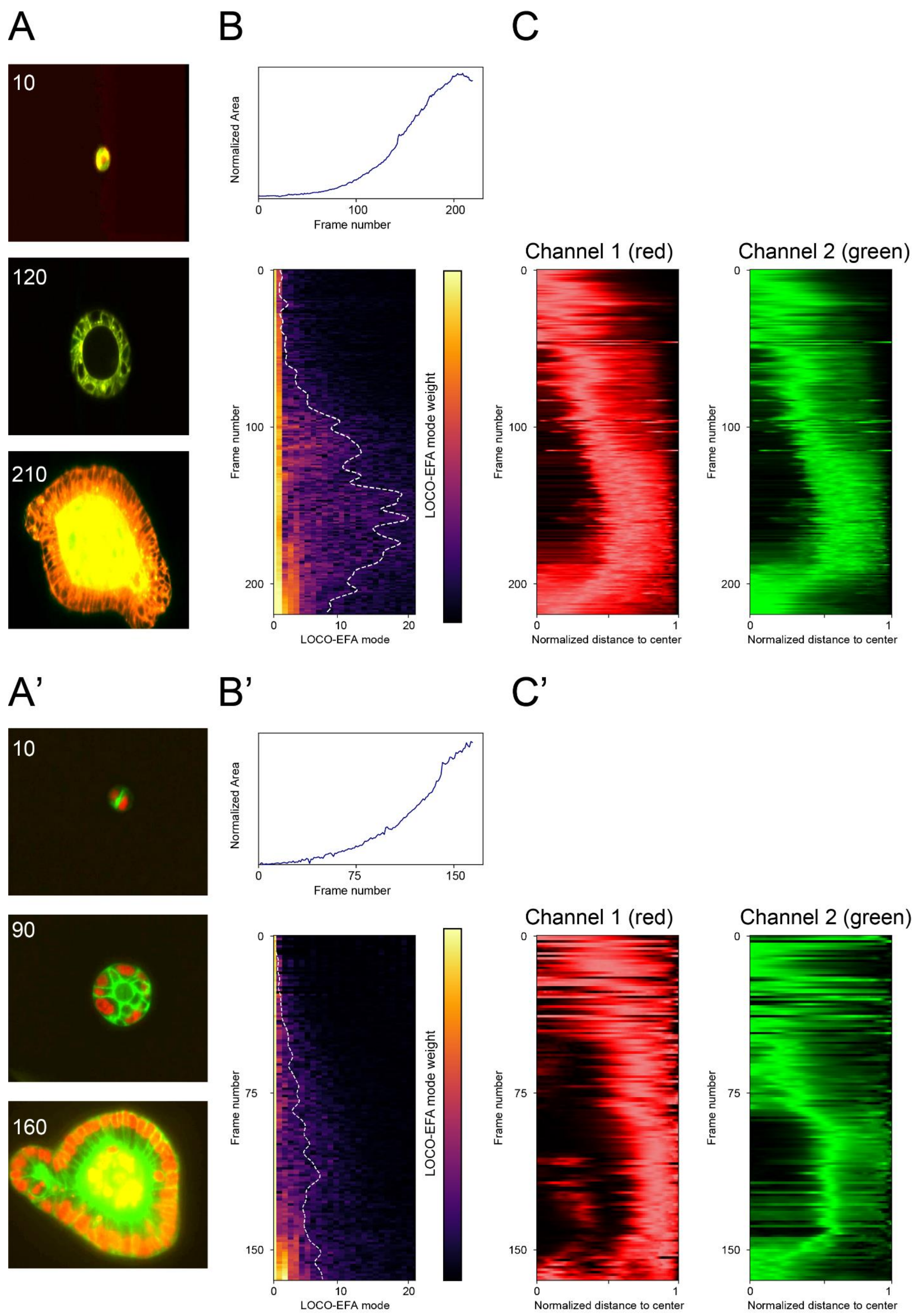

Figure S5. Quantitative analysis of intestinal organoid development. A, A') Representative images of two time lapse datasets of intestinal organoids adapted from (Serra et al. 2019). White texts indicate frame number in the time lapse. B,B') Morphology quantification of the time lapse datasets. Top: Area normalized by the area at the first time point. Bottom: LOCO-EFA coefficients for each individual image in the datasets. White dashed lines represent the highest elliptical mode necessary to describe the organoid shape with a 95% accuracy. C, C') Kymograph of the radial profiles for the two channels in the raw images. Red: ubiquitous cell membrane (top) and nuclear marker expression (bottom dataset). Green: Expression of the stem cell marker LGR5 (top) and membrane marker (bottom).

Development • Supplementary information

Table S1

Figure	Sample	Imaging modality	Pixel size (x,y) (um)	Frame size (pixels)
Fig 2 A-D	Human brain organoid	Confocal	2.5	~500 x 250
Fig 2 E-G	Pescoids	Dissection microscope	1.25	~1000 x 500
Fig 3	Gastruloids	HCS	0.6	2160 x 2160
Sup Fig 3	Intestinal organoids	Light Sheet Fluorescence Microscope	0.12	~800 x 800

Development • Supplementary information
